# Knowledge of Parkinson’s disease among non-PD specialists: a web-based survey in South China

**DOI:** 10.3389/fnagi.2025.1488009

**Published:** 2025-04-09

**Authors:** Shaohua Lyu, Zhe Li, Zhenhui Mao, Jingbo Sun, Chunye Zheng, Qiaozhen Su

**Affiliations:** The Second Affiliated Hospital of Guangzhou University of Chinese Medicine, Guangdong Provincial Hospital of Chinese Medicine and Guangdong Provincial Academy of Chinese Medical Sciences, Guangzhou, China

**Keywords:** Parkinson’s disease, web-based survey, motor symptoms, non-motor symptoms, prodromal symptoms, risk factors, antiparkinsonian medications

## Abstract

**Background:**

Parkinson’s disease (PD) is a prevalent, disabling neurodegenerative disorder. Early diagnosis and treatment of PD remains challenging due to the absence of definitive diagnostic tests and the non-specificity of its clinical manifestations. Initial consultations for PD symptoms often involve specialists who are not specifically trained in PD. Consequently, it is imperative to assess the general knowledge regarding PD among these specialists to develop optimal educational strategies and enhance early recognition and diagnosis of PD.

**Methods:**

We developed a questionnaire covering motor symptoms, non-motor symptoms, prodromal symptoms, risk factors and antiparkinsonian medications based on published guidelines, and conducted the web-based survey via *Wenjuan xing* (https://www.wjx.cn/) among physicians not specializing in PD in Guangdong Province, China.

**Results:**

A total of 312 respondents, working in 28 diverse departments across 64 hospitals of three different categories, were eligible for data analysis. Notably, 95.2% of the respondents were aware of rest tremor as a motor symptom, yet only 76.9% recognized bradykinesia as a motor symptom. Regarding non-motor symptoms, erectile dysfunction, urinary dysfunction, restless legs, olfactory loss, orthostatic hypotension, rapid eye movement behavior disorder (RBD), lower back pain and diaphoresis, were recognized by less than 50% of the respondents. Additionally, with the exception of subthreshold parkinsonism or abnormal quantitative motor testing, prodromal symptoms such as excessive daytime somnolence, depression (± anxiety), olfactory loss, urinary dysfunction, RBD, and constipation were recognized by 36.5–48.7% of the respondents. First-degree relatives with PD received recognition from 86.5% of the respondents, whereas the remaining risk factors were recognized by 50–60% of the participants. Concerning protective factors for PD, recognition was limited to no more than 23%. Levodopa and dopamine releasers were the most widely recognized antiparkinsonian medications, while the recognition of other medications was below 70%. Variables such as medical degrees, professional titles, hospital categories, and education subjects contributed to statistical differences in PD knowledge.

**Conclusion:**

Among non-PD specialists in south China, current knowledge regarding PD, including non-motor symptoms, prodromal symptoms, risk and protective factors, and antiparkinsonian medications, is relatively inadequate. This necessitates targeted education and training to improve their understanding and recognition of PD.

## 1 Introduction

Parkinson’s disease (PD) is a prevalent neurodegenerative condition, characterized by typical motor symptoms, such as akinesia, bradykinesia, tremor, rigidity, and postural instability. Additionally, patients with PD often exhibit various non-motor symptoms including constipation, olfactory dysfunction, depression, etc. (Postuma et al., 2015). Globally, PD affects an estimated 8.5 million individuals, with a reported age-standardized rate of prevalence at 0.106% in 2019, making it the second most common neurodegenerative disease after Alzheimer’s Disease (Ou et al., 2021). Furthermore, in 2019, PD led to a global burden of 1.21 × 10^6^ years lived with disability (Ou et al., 2021). In China specifically, the age-standardized incidence and prevalence rates for PD reached 2.43‰ and 2.457‰ in 2021, exceeding the global average and surpassing those of other G20 countries. Notably, the incidence and prevalence of PD in the southeast coastal regions of China were disproportionately higher than in other regions within the country (Xu et al., 2024).

Diagnosis of PD relies heavily on clinical manifestations and there are no definitive examinations (Postuma et al., 2015). Despite ongoing updates to diagnostic criteria, the accuracy of PD diagnosis is limited, ranging from 73.8 to 83.9% (Rizzo et al., 2016). Moreover, in prodromal or early stages of PD when classical motor symptoms have not been manifested, non-motor symptoms may become leading causes of hospital visits. However, the non-specificity of non-motor symptoms significantly results in misdiagnosis or underdiagnosis of PD in clinical practice (Greenland and Barker, 2018). Notably, a significant number of patients worldwide seek diagnosis from physicians not specialized in PD or movement disorders (Lim et al., 2022; 赵亚军, 龚继章, 葛许华, 2022), leading to a higher rate of PD diagnosis compared to specialists in this field (Tinelli et al., 2016; Virameteekul et al., 2023). Furthermore, PD diagnosis is typically confirmed when classical motor symptoms appear in a stage with substantial neurophysiological damage, at which point approximately 70% of dopamine neurons may have been lost (Murman, 2012). Intervention at this stage is often too late to delay disease progression or achieve neuroprotection. Therefore, early and timely diagnosis of PD based on non-motor symptoms in prodromal stage presents a valuable opportunity for early therapeutic intervention to alleviate symptoms, slow disease progression, improve quality of life and reduce long-term costs (Pagan, 2012; Soman et al., 2023; Tinelli et al., 2016).

Antiparkinsonian medications, including levodopa, dopamine agonists and monoamine oxidase type-B (MAO-B) inhibitors, etc., can significantly alleviate motor symptoms. Surprisingly, 76.7% of general practitioners (GPs) are not comfortable initiating antiparkinsonian medications primarily due to unfamiliarity with these medications (Lim et al., 2022). Notably, the exclusive dosage and duration of levodopa treatment may contribute to the occurrence of motor complications (Sun et al., 2020). Therefore, there is a need for preceding investigation into the general knowledge of antiparkinsonian medications before developing an educational strategy on the appropriate timing and strategy for prescribing antiparkinsonian medications.

In China, both self-referral and GP-referral are available, resulting in non-PD specialists take significantly active involvement in the diagnosis and management of PD (Heping, 2021; Li et al., 2019). Consequently, early recognition and diagnosis of PD are challenging in real-world clinical practice in China. Additionally, healthcare system in China comprises a tertiary network, consisting of primary, secondary, and tertiary hospitals. Community-based primary hospitals and GPs, provide basic services for the prevention, diagnosis, and treatment of common conditions. Secondary hospitals cater to a larger number of communities with comprehensive medical care, while tertiary hospitals offer specialized care for complicated cases (Heping, 2021). It has been reported that education and training of primary healthcare practitioners are inadequate (Li et al., 2020), highlighting the necessity to further explore the knowledge of PD among non-specialist healthcare professionals and promote PD education to reduce the potential misdiagnosis of PD. A comprehensive survey of PD knowledge among non-specialists healthcare professionals across various categories of hospitals would provide guidance for optimizing an education strategy and improving the quality of training for these physicians (Li et al., 2020).

Consequently, we designed a cross-sectional survey targeting physicians who are not specializing in PD. The objective of this survey was to evaluate the general knowledge of PD among non-PD specialists and to assess the barriers that hinder early diagnosis and appropriate treatment of PD in China.

## 2 Methods

The survey was conducted anonymously among non-PD specialists residing in Guangdong Province, China, utilizing a structured online questionnaire. The findings of this survey are reported in strict adherence to the Consensus-Based Checklist for Reporting of Survey Studies guidelines (Sharma et al., 2021).

### 2.1 Questionnaire development

The structured questionnaire was meticulously developed to investigate physicians’ knowledge pertaining to motor symptoms, non-motor symptoms, prodromal symptoms, risk factors and commonly prescribed antiparkinsonian medications.

The questionnaire was grounded in PD clinical diagnostic criteria (Clarke et al., 2016; Parkinson's Disease and movement Disorders Group NB, Chinese Medical Association, Parkinson's Disease and movement Disorders Professional Committee of Neurology Branch of Chinese Medical Doctor Association, 2016), research criteria for PD prodromal symptoms (Heinzel et al., 2019; 陈彪, 陈生弟, 刘疏影, 2019), treatment guidelines for PD (Grimes et al., 2019; Health NIf, Excellence C, 2017; 中华医学会神经病学分会帕金森病及运动障碍学组, 中国医师协会神经内科医师分会帕金森病及运动障碍学组, 2020), and related scholarly works (Kayis et al., 2023; Sun et al., 2020). It was initially drafted by Shaohua Lyu, with 56 questions covering demographic information, knowledge of PD involving motor symptoms, non-motor symptoms, prodromal symptoms, risk factors, protective factors and antiparkinsonian medications, as well as preferred treating methods in real-world clinical practice. Subsequently, it underwent a thorough review and approval process by five PD experts from Guangdong Provincial Hospital of Chinese Medicine (GPHCM). Based on the feedback collected from a pre-testing pilot survey involving five non-PD physicians from GPHCM, language was polished to be plain and enhance the clarity of the items. Redundant questions were condensed for conciseness and questions related with real-world treating methods were removed from the final version as it is beyond the frame of research aim.

The final version comprises 44 questions specifically tailored to PD, respondents were asked to judge whether a given symptom belongs to motor symptom, non-motor symptom, prodromal symptom, risk and protective factor of PD, or a medicine belongs to antiparkinsonian medications (see Supplementary material 1 for more details). The questionnaire was made available as an electronic questionnaire on *Wenjuan xing*,^1^ a widely utilized online survey platform in China. This platform offers convenient access for data collection. Additionally, logic rules were carefully designed and implemented to streamline the question-answering process, compulsory response to each question was also implemented to minimize incomplete data.

The questionnaire can be accessed via https://www.wjx.cn/vm/mQJ5kge.aspx#.

### 2.2 Sample characteristics

Clinicians who do not specialize in PD and are employed within Guangdong Province were eligible to participate in the survey, regardless of their hospital category, department, educational qualifications, subjects studied, or professional titles. However, clinicians specializing in PD, nurses, physicians not actively engaged in clinical practice, and those not residing in Guangdong Province were excluded from participation.

Convenience sampling was employed to identify potential respondents during the survey. The minimum sample size was calculated to be 273, with a 90% confidence interval and a 5% error margin by the below formular:


$$n=\frac{Z^{2}\times p\times \left(1-p\right)}{E^{2}}$$

Where *n* is the sample size, *Z* is the Z-score, *p* is the estimated proportion of an attribute that is present in the population, and *E* is the margin of error.

### 2.3 Survey administration

The online survey was actively promoted among the memberships of Guangdong Association of Integrative Chinese and Western Medicine, Guangdong Association of Traditional Chinese Medicine, and Guangdong Association of Medicine from November to December 2023. To ensure the accuracy and uniqueness of responses, measures were taken during the design stage to prevent duplicate or multiple participation through the incorporation of logic checks. Furthermore, additional data-checking was conducted to eliminate any potential instances of multiple participation, thereby safeguarding the integrity of the survey results.

### 2.4 Ethical considerations

The study was exempted from ethical review by the ethics committee of GPHCM (ZM2023-393), and conducted anonymously, adhering strictly to the principles outlined in the Helsinki Declaration (Goodyear et al., 2007). Confidential information, including names, contact details, and addresses, was not collected during the survey, ensuring the privacy and safety of all participants.

### 2.5 Statistical methods

SPSS 26 (Corp, 2017) was utilized for data analysis. Continuous data were described using the mean and standardized deviation, and further comparisons were conducted using the Student’s t-test, where applicable. Categorical data was described by frequency and percentage, and further compared using the chi-square test. Furthermore, multiple regression analyses were conducted to identify demographic factors that result in differences of knowledge of PD.

## 3 Results

### 3.1 Summary of the research procedure

**Figure 1:**
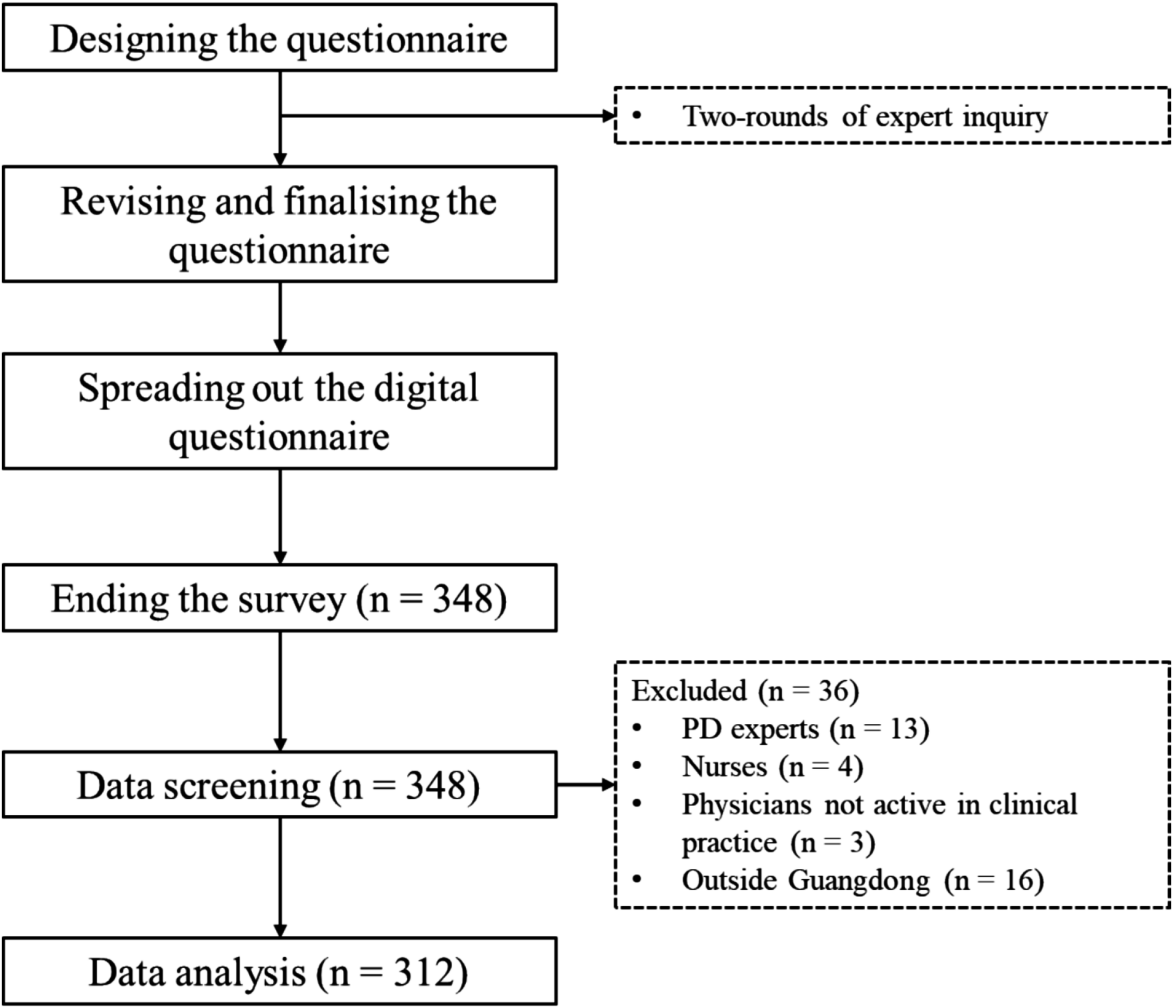
Flowchart of the study.

Initially, 348 physicians responded to our survey and completed the online questionnaire, and 312 questionnaires were deemed eligible for data analyses after throughout screening (Figure 1).

### 3.2 Clinical features of all respondents

#### 3.2.1 Demographics and general characteristics

The survey comprises 312 respondents from 64 hospitals across 17 cities in Guangdong Province, with a mean age of 37.59 ± 6.82 years and 12.04 ± 7.74 years of clinical experience. The majority practiced in tertiary hospitals (74.36%), spanning 28 departments (93.27% non-neurology). Academic qualifications included master’s (52.56%), bachelor’s or lower (40.06%), and doctoral degrees (7.37%). Medical training backgrounds encompassed Chinese (41.67%), conventional (25.64%), and integrated medicine (32.69%). Professional titles comprised attending (41.67%), associate chief/chief (33.33%), and resident physicians (25%) (Supplementary file 2).

#### 3.2.2 Knowledge on PD symptoms

Table 1 provides a comprehensive overview of participants’ responses to motor, non-motor and prodromal symptoms, as well as risk factors and antiparkinsonian medications. Notably, rest tremor was the most widely recognized motor symptom (*n* = 297, 95.2%), followed by postural instability (*n* = 280, 89.7%) and rigidity (*n* = 275, 88.1%). Bradykinesia was the least acknowledged (*n* = 240, 76.9%).

**Table 1:** Responses to motor, non-moto and prodromal symptoms, risk factors, and antiparkinsonian medications.

Item		Frequency of correct response (%)	Reported prevalence
Motor symptom	Tremor	297 (95.2%)	79.9%–96.2%
	Postural instability	280 (89.7%)	16%
	Rigidity	275 (88.1%)	89%
	Bradykinesia	240 (76.9%)	65%
Non-motor symptoms	Global cognitive deficit	225 (72.1%)	40%–65.82%
	Constipation	194 (62.2%)	50%–64.56%
	Sialorrhea	185 (59.3%)	31%–56%
	Depression and/or anxiety	176 (56.4%)	56%
	Fatigue	173 (55.4%)	36.8%–58%
	Insomnia	172 (55.1%)	37%–83%
	Erectile dysfunction	154 (49.4%)	57.6%–79%
	Urinary dysfunction	151 (48.4%)	39.3%–87.8%
	Restless legs	146 (46.8%)	14%
	Olfactory loss	141 (45.2%)	65.0%–75.5%
	Orthostatic hypotension	119 (38.1%)	30.1%
	Rapid eye movement behavior disorder	112 (35.9%)	37%–42.3%
	Lower back pain	112 (35.9%)	42.5%–83%
	Diaphoresis (Excessive sweating)	94 (30.1%)	64%

Item		Frequency of correct response (%)	Reported prevalence	Positive likelihood ratio of risk and prodromal markers
Prodromal symptoms	Subthreshold parkinsonism or abnormal quantitative motor testing	209 (67.0%)	NA	3.5–9.6
	Excessive daytime somnolence	152 (48.7%)	65.63%	2.7
	Depression (± anxiety)	148 (47.4%)	12.0%, anxiety 8.8	1.6
	Olfactory loss	133 (42.6%)	3.9%	6.4
	Urinary dysfunction	131 (42.0%)	53.13%	2.0
	Rapid eye movement behavior disorder	128 (41.0%)	13.3%	2.8–130
	Constipation	127 (40.7%)	9.7%	2.5
	Orthostatic hypotension	120 (38.5%)	18.2	18.5
	Erectile dysfunction	114 (36.5%)	20.8%	3.4 (in men)

Item		Frequency of correct response (%)	Positive likelihood ratio of risk and prodromal markers
Risk factors	First-degree relative with PD	270 (86.5%)	2.5
	Occupational solvent exposure	190 (60.9%)	1.5
	Diabetes mellitus (type II)	174 (55.8%)	1.5
	Regular pesticide exposure	165 (52.9%)	1.5
	Male sex	161 (51.6%)	1.2
	Physical inactivity	156 (50.0%)	1.3
	Intake of tea^#^	71 (22.8%)	0.55*
	Intake of caffeine^#^	44 (14.1%)	0.88*
	Smoking^#^	9 (2.9%)	0.51–0.91*
	Low plasma urate levels	5 (1.6%)	1.9

Item		Frequency of correct response (%)
Antiparkinsonian medications	Levodopa	248 (79.5%)
	Dopamine releasers	239 (76.6%)
	Anticholinergics	216 (69.2%)
	Dopamine agonists	205 (65.7%)
	Levodopa decarboxylase inhibitors	179 (57.4%)
	MAO-B inhibitors	158 (50.6%)
	COMT inhibitors	148 (47.4%)

^#Protective factors; negative likelihood ratio.^

Among the reported non-motor symptoms, global cognitive deficit ranked highest with 225 (72.1%) respondents recognizing it. Other commonly recognized symptoms include constipation (*n* = 192, 62.2%), sialorrhea (*n* = 185, 59.3%), anxiety (*n* = 176, 56.4%), fatigue (*n* = 173, 55.4%) and insomnia (*n* = 172, 55.1%). Notably, less than half of the respondents recognized non-motor symptoms such as erectile dysfunction, urinary dysfunction, restless legs, olfactory loss, orthostatic hypotension, RBD, lower back pain and diaphoresis.

Subthreshold parkinsonism or abnormal quantitative motor testing emerged as the sole prodromal symptom recognized by over 50% of respondents. Other prodromal features including excessive daytime somnolence (*n* = 152, 48.7%), depression (± anxiety) (*n* = 148, 47.4%), olfactory loss (*n* = 133, 42.6%), urinary dysfunction (*n* = 131, 42.0%), RBD (*n* = 128, 41%), and constipation (*n* = 127, 40.7%), were recognized by <50%.

First-degree familial PD history was the most recognized risk factor 270 (86.5%). Other risk factors recognized by over 50% of respondents include occupational solvent exposure (*n* = 190, 60.9%), diabetes mellitus (*n* = 174, 55.8%), regular pesticide exposure (*n* = 165, 52.9%), male sex (*n* = 161, 51.6%) and physical inactivity (*n* = 156, 50%). Prospective factors, like smoking, intake of tea and caffeine, were less recognized by respondents, with only 71 (22.8%), 44 (14.1%) and 9 (2.9%) respondents recognizing them, respectively. Notably, a mere 5 (1.6%) respondents identified low plasma urate level as a risk factor for PD.

In terms of antiparkinsonian medications commonly used in current clinical practice, levodopa stands out as the most familiar to respondents, with 248 (79.5%) acknowledging it. Dopamine releasers (*n* = 239, 76.6%), anticholinergics (*n* = 216, 69.2%), and dopamine agonists (*n* = 205, 65.7%) also enjoy widespread recognition. Conversely, MAO-B inhibitors and catechol-O-methyltransferase (COMT) inhibitors are less familiar by the respondents, with frequencies of 158 (50.6%) and 148 (47.4%), respectively.

#### 3.2.3 Comparisons of PD knowledge

Comparison of PD knowledge regarding motor, non-motor and prodromal symptoms, risk factors and antiparkinsonian medications was conducted across education qualifications, professional titles, categories of hospitals and education subjects. The findings of these comparisons are presented in Tables 2–5, respectively.

**Table 2:** Comparisons among education qualifications.

Item		Responses	Education qualifications			Statistical difference	
			Bachelor’s degree in medicine or below (*n* = 125)	Master’s degree in medicine (*n* = 164)	Doctoral degree in medicine (*n* = 23)	Value	*p*
Motor symptom	Tremor	Yes	116 (92.8%)_a_	159 (97.0%)_a_	22 (95.7%)_a_	2.692	0.239
		No	9 (7.2%)_a_	5 (3.0%)_a_	1 (4.3%)_a_		
	Postural instability	Yes	110 (88.0%)_a_	150 (91.5%)_a_	20 (87.0%)_a_	1.386	0.538
		No	15 (12.0%)_a_	14 (8.5%)	3 (13.0%)		
	Rigidity	Yes	107 (85.6%)_a_	146 (89.0%)_a_	22 (95.7%)_a_	1.798	0.406
		No	18 (14.4%)_a_	18 (11.0%)_a_	1 (4.3%)_a_		
	Bradykinesia	Yes	93 (74.4%)_a_	128 (78.0%)_a_	19 (82.6%)_a_	0.877	0.663
		No	32 (25.6%)_a_	36 (22.0%)_a_	4 (17.4%)_a_		
Non-motor symptoms	Global cognitive deficit	Yes	85 (68.0%)_a_	124 (75.6%)_a_	16 (69.6%)_a_	2.198	0.341
		No	40 (32.0%)_a_	40 (24.4%)_a_	7 (30.4%)_a_		
	Constipation	Yes	77 (61.6%)_a_	100 (61.0%)_a_	17 (73.9%)_a_	1.394	0.520
		No	48 (38.4%)_a_	64 (39.0%)_a_	6 (26.1%)_a_		
	Sialorrhea	Yes	72 (57.6%)_a_	101 (61.6%)_a_	12 (52.2%)_a_	1.036	0.605
		No	53 (42.4%)_a_	63 (38.4%)_a_	11 (47.8%)_a_		
	Depression (± anxiety)	Yes	73 (58.4%)_a_	90 (54.9%)_a_	13 (56.5%)_a_	0.381	0.848
		No	52 (41.6%)_a_	74 (45.1%)_a_	10 (43.5%)_a_		
	Fatigue	Yes	70 (56.0%)_a_	93 (56.7%)_a_	10 (43.5%)_a_	1.458	0.494
		No	55 (44.0%)_a_	71 (43.3%)_a_	13 (56.5%)_a_		
	Insomnia	Yes	72 (57.6%)_a_	91 (55.5%)_a_	9 (39.1%)_a_	2.663	0.269
		No	53 (42.4%)_a_	73 (44.5%)_a_	14 (60.9%)_a_		
	Erectile dysfunction	Yes	60 (48.0%)_a_	85 (51.8%)_a_	9 (39.1%)_a_	1.442	0.467
		No	65 (52.0%)_a_	79 (48.2%)_a_	14 (60.9%)_a_		
	Urinary dysfunction	Yes	62 (49.6%)_a_	80 (48.8%)_a_	9 (39.1%)_a_	0.863	0.646
		No	63 (50.4%)_a_	84 (51.2%)_a_	14 (60.9%)_a_		
	Restless legs	Yes	57 (45.6%)_a_	79 (48.2%)_a_	10 (43.5%)_a_	0.312	0.892
		No	68 (54.4%)_a_	85 (51.8%)_a_	13 (56.5%)_a_		
	Olfactory loss	Yes	62 (49.6%)_a_	70 (42.7%)_a_	9 (39.1%)_a_	1.726	0.426
		No	63 (50.4%)_a_	94 (57.3%)_a_	14 (60.9%)_a_		
	Orthostatic hypotension	Yes	42 (33.6%)_a_	64 (39.0%)_a_	13 (56.5%)_a_	4.362	0.114
		No	83 (66.4%)_a_	100 (61.0%)_a_	10 (43.5%)_a_		
	Lower back pain	Yes	43 (34.4%)_a_	61 (37.2%)_a_	8 (34.8%)_a_	0.267	0.916
		No	82 (65.6%)_a_	103 (62.8%)_a_	15 (65.2%)_a_		
	Rapid eye movement behavior disorder	Yes	41 (32.8%)_a_	63 (38.4%)_a_	8 (34.8%)_a_	0.989	0.623
		No	84 (67.2%)_a_	101 (61.6%)_a_	15 (65.2%)_a_		
	Diaphoresis (Excessive sweating)	Yes	38 (30.4%)_a_	48 (29.3%)_a_	8 (34.8%)_a_	0.387	0.858
		No	87 (69.6%)_a_	116 (70.7%)_a_	15 (65.2%)_a_		
Prodromal symptoms	Subthreshold parkinsonism or abnormal quantitative motor testing	Yes	89 (68.5%)_a_	52 (65.0%)_a_	68 (66.7%)_a_	0.3	0.853
		No	41 (31.5%)_a_	28 (35.0%)_a_	34 (33.3%)_a_		
	Excessive daytime somnolence	Yes	68 (52.3%)_a_	37 (46.3%)_a_	47 (46.1%)_a_	1.152	0.569
		No	62 (47.7%)_a_	43 (53.8%)_a_	55 (53.9%)_a_		
	Depression (± anxiety)	Yes	68 (52.3%)_a_	35 (43.8%)_a_	45 (44.1%)_a_	2.114	0.343
		No	62 (47.7%)_a_	45 (56.3%)_a_	57 (55.9%)_a_		
	Olfactory loss	Yes	57 (43.8%)_a_	34 (42.5%)_a_	42 (41.2%)_a_	0.177	0.905
		No	73 (56.2%)_a_	46 (57.5%)_a_	60 (58.8%)_a_		
	Urinary dysfunction	Yes	60 (46.2%)_a_	28 (35.0%)_a_	43 (42.2%)_a_	2.522	0.28
		No	70 (53.8%)_a_	52 (65.0%)_a_	59 (57.8%)_a_		
	Rapid eye movement behavior disorder	Yes	57 (43.8%)_a_	28 (35.0%)_a_	43 (42.2%)_a_	1.679	0.436
		No	73 (56.2%)_a_	52 (65.0%)_a_	59 (57.8%)_a_		
	Constipation	Yes	61 (46.9%)_a_	23 (28.8%)_b_	43 (42.2%)_a,b_	6.981	0.029*
		No	69 (53.1%)_a_	57 (71.3%)_b_	59 (57.8%)_a,b_		
	Orthostatic hypotension	Yes	53 (40.8%)_a_	26 (32.5)_a_	41 (40.2%)_a_	1.622	0.448
		No	77 (59.2%)_a_	54 (67.5%)_a_	61 (59.8%)_a_		
	Erectile dysfunction	Yes	48 (36.9%)_a_	32 (40.0%)_a_	34 (33.3%)_a_	0.886	0.634
		No	82 (63.1%)_a_	48 (60.0%)_a_	68 (66.7%)_a_		
Risk factors	First-degree relative with PD	Yes	115 (88.5%)_a_	68 (85.0%)_a_	87 (85.3%)_a_	0.785	0.676
		No	15 (11.5%)_a_	12 (15.0%)_a_	15 (14.7%)_a_		
	Occupational solvent exposure	Yes	76 (58.5%)_a_	53 (66.3%)_a_	61 (59.8%)_a_	1.334	0.522
		No	54 (41.5%)_a_	27 (33.8%)_a_	41 (40.2%)_a_		
	Diabetes mellitus (type II)	Yes	79 (60.8%)_a_	49 (61.3%)_a_	46 (45.1%)_b_	6.927	0.032*
		No	51 (39.2%)_a_	31 (38.8%)_a_	56 (54.9%)_b_		
	Regular pesticide exposure	Yes	71 (54.6%)_a_	43 (53.8%)_a_	51 (50.0%)_a_	0.53	0.787
		No	59 (45.4%)_a_	37 (46.3%)_a_	51 (50.0%)_a_		
	Male sex	Yes	71 (54.6%)_a_	44 (55.0%)_a_	46 (45.1%)_a_	2.56	0.283
		No	59 (45.4%)_a_	36 (45.0%)_a_	56 (54.9%)_a_		
	Physical inactivity	Yes	60 (46.2%)_a_	43 (53.8%)_a_	53 (52.0%)_a_	1.378	0.516
		No	70 (53.8%)_a_	37 (46.3%)_a_	49 (48.0%)_a_		
	Intake of tea^#^	Yes	28 (21.5%)_a_	24 (30.0%)_a_	19 (18.6%)_a_	3.395	0.179
		No	102 (78.5%)_a_	56 (70.0%)_a_	83 (81.4%)_a_		
	Intake of caffeine^#^	Yes	14 (10.8%)_a_	15 (18.8%)_a_	15 (14.7%)_a_	2.69	0.255
		No	116 (89.2%)_a_	65 (81.3%)_a_	87 (85.3%)_a_		
	Smoking^#^	Yes	4 (3.1%)_a_	2 (2.5%)_a_	3 (2.9%)_a_	0.166	1
		No	126 (96.9%)_a_	78 (97.5%)_a_	99 (97.1%)_a_		
	Low plasma urate levels	Yes	4 (3.1%)_a_	0 (0.0%)_a_	1 (1.0%)_a_	2.542	0.318
		No	126 (96.9%)_a_	80 (100.0%)_a_	101 (99.0%)_a_		
Antiparkinsonian medications	Levodopa	Yes	104 (80.0%)_a_	65 (81.3%)_a_	79 (77.5%)_a_	0.435	0.82
		No	26 (20.0%)_a_	15 (18.8%)_a_	23 (22.5%)_a_		
	Dopamine releasers	Yes	93 (71.5%)_a_	66 (82.5%)_a_	80 (78.4%)_a_	3.506	0.172
		No	37 (28.5%)_a_	14 (17.5%)_a_	22 (21.6%)_a_		
	Anticholinergics	Yes	82 (63.1%)_a_	61 (76.3%)_a_	73 (71.6%)_a_	4.343	0.111
		No	48 (36.9%)_a_	19 (23.8%)_a_	29 (28.4%)_a_		
	Dopamine agonists	Yes	86 (66.2%)_a_	52 (65.0%)_a_	67 (65.7%)_a_	0.048	0.988
		No	44 (33.8%)_a_	28 (35.0%)_a_	35 (34.3%)_a_		
	Levodopa decarboxylase inhibitors	Yes	71 (54.6%)_a_	52 (65.0%)_a_	56 (54.9%)_a_	2.56	0.276
		No	59 (45.4%)_a_	28 (35.0%)_a_	46 (45.1%)_a_		
	MAO-B inhibitors	Yes	67 (51.5%)_a_	45 (56.3%)_a_	46 (45.1%)_a_	2.29	0.31
		No	63 (48.5%)_a_	35 (43.8%)_a_	56 (54.9%)_a_		
	COMT inhibitors	Yes	61 (46.9%)_a_	39 (48.8%)_a_	48 (47.1%)_a_	0.088	0.968
		No	69 (53.1%)_a_	41 (51.3%)_a_	54 (52.9%)_a_		

Each subscript letter denotes a subset of education qualification categories whose column proportions do not differ significantly from each other at the 0.05 level. ^#^Protective factor for PD. *Significant difference at 0.05 level. COMT, catechol-O-methyltransferase; MAO-B, monoamine oxidase-B; PD, Parkinson’s disease.

**Table 3:** Comparisons among professional titles.

Item		Response	Professional title			Statistical difference	
			Associate Chief physicians or Chief physicians (*n* = 104)	Attending physicians (*n* = 130)	Resident physicians (*n* = 78)	Value	*p*
Motor symptom	Tremor	Yes	99 (95.2%)_a_	126 (96.9%)_a_	72 (92.3%)_a_	2.282	0.305
		No	5 (4.8%)_a_	4 (3.1%)_a_	6 (7.7%)_a_		
	Postural instability	Yes	94 (90.4%)_a_	119 (91.5%)_a_	67 (85.9%)_a_	1.752	0.441
		No	10 (9.6%)	11 (8.5%)	11 (14.1%)		
	Rigidity	Yes	96 (92.3%)_a_	114 (87.7%)_a_	65 (83.3%)_a_	3.494	0.173
		No	8 (7.7%)_a_	16 (12.3%)_a_	13 (16.7%)_a_		
	Bradykinesia	Yes	87 (83.7%)_a_	101 (77.7%)_a,b_	52 (66.7%)_b_	7.115	0.028*
		No	17 (16.3%)_a_	29_a_, (22.3%)_b_	26 (33.3%)_b_		
Non-motor symptoms	Global cognitive deficit	Yes	77 (74.0%)_a_	97 (74.6%)_a_	51 (65.4%)_a_	2.319	0.312
		No	27 (26.0%)_a_	33 (25.4%)_a_	27 (34.6%)_a_		
	Constipation	Yes	65 (62.5%)_a_	87 (66.9%)_a_	42 (53.8%)_a_	3.527	0.168
		No	39 (37.5%)_a_	43 (33.1%)_a_	36 (46.2%)_a_		
	Sialorrhea	Yes	64 (61.5%)_a_	78 (60.0%)_a_	43 (55.1%)_a_	0.814	0.682
		No	40 (38.5%)_a_	52 (40.0%)_a_	35 (44.9%)_a_		
	Depression (± anxiety)	Yes	58 (55.8%)_a_	73 (56.2%)_a_	45 (57.7%)_a_	0.084	0.978
		No	46 (44.2%)_a_	57 (43.8%)_a_	33 (42.3%)_a_		
	Fatigue	Yes	56 (53.8%)_a_	76 (58.5%)_a_	41 (52.6%)_a_	0.862	0.651
		No	48 (46.2%)_a_	54 (41.5%)_a_	37 (47.4%)_a_		
	Insomnia	Yes	53 (51.0%)_a_	76 (58.5%)_a_	43 (55.1%)_a_	1.319	0.511
		No	51 (49.0%)_a_	54 (41.5%)_a_	35 (44.9%)_a_		
	Erectile dysfunction	Yes	52 (50.0%)_a_	69 (53.1%)_a_	33 (42.3%)_a_	2.281	0.325
		No	52 (50.0%)_a_	61 (46.9%)_a_	45 (57.7%)_a_		
	Urinary dysfunction	Yes	46 (44.2%)_a_	69 (53.1%)_a_	36 (46.2%)_a_	2.015	0.359
		No	58 (55.8%)_a_	61 (46.9%)_a_	42 (53.8%)_a_		
	Restless legs	Yes	51 (49.0%)_a_	56 (43.1%)_a_	39 (50.0%)_a_	1.262	0.548
		No	53 (51.0%)_a_	74 (56.9%)_a_	39 (50.0%)_a_		
	Olfactory loss	Yes	48 (46.2%)_a_	58 (44.6%)_a_	35 (44.9%)_a_	0.073	0.978
		No	56 (53.8%)_a_	72 (55.4%)_a_	43 (55.1%)_a_		
	Orthostatic hypotension	Yes	42 (40.4%)_a_	52 (40.0%)_a_	25 (32.1%)_a_	1.637	0.451
		No	62 (59.6%)_a_	78 (60.0%)_a_	53 (67.9%)_a_		
	Lower back pain	Yes	42 (40.4%)_a_	45 (34.6%)_a_	25 (32.1%)_a_	1.487	0.469
		No	62 (59.6%)_a_	85 (65.4%)_a_	53 (67.9%)_a_		
	Rapid eye movement behavior disorder	Yes	36 (34.6%)_a_	45 (34.6%)_a_	31 (39.7%)_a_	0.684	0.727
		No	68 (65.4%)_a_	85 (65.4%)_a_	47 (60.3%)_a_		
	Diaphoresis (Excessive sweating)	Yes	29 (27.9%)_a_	39 (30.0%)_a_	26 (33.3%)_a_	0.649	0.734
		No	75 (72.1%)_a_	91 (70.0%)_a_	52 (66.7%)_a_		
Prodromal symptoms	Subthreshold parkinsonism or abnormal quantitative motor testing	Yes	68 (65.4%)_a_	86 (66.2%)_a_	55 (70.5%)_a_	0.598	0.745
		No	36 (34.6%)_a_	44 (33.8%)_a_	23 (29.5%)_a_		
	Excessive daytime somnolence	Yes	50 (48.1%)_a_	59 (45.4%)_a_	43 (55.1%)_a_	1.875	0.392
		No	54 (51.9%)_a_	71 (54.6%)_a_	35 (44.9%)_a_		
	Depression (± anxiety)	Yes	51 (49.0%)_a_	57 (43.8%)_a_	40 (51.3%)_a_	1.248	0.537
		No	53 (51.0%)_a_	73 (56.2%)_a_	38 (48.7%)_a_		
	Olfactory loss	Yes	49 (47.1%)_a_	51 (39.2%)_a_	33 (42.3%)_a_	1.476	0.485
		No	55 (52.9%)_a_	79 (60.8%)_a_	45 (57.7%)_a_		
	Urinary dysfunction	Yes	47 (45.2%)_a_	55 (42.3%)_a_	29 (37.2%)_a_	1.182	0.553
		No	57 (54.8%)_a_	75 (57.7%)_a_	49 (62.8%)_a_		
	Rapid eye movement behavior disorder	Yes	43 (41.3%)_a_	46 (35.4%)_a_	39 (50.0%)_a_	4.289	0.12
		No	61 (58.7%)_a_	84 (64.6%)_a_	39 (50.0%)_a_		
	Constipation	Yes	48 (46.2%)_a_	52 (40.0%)_a_	27 (34.6%)_a_	2.486	0.292
		No	56 (53.8%)_a_	78 (60.0%)_a_	51 (65.4%)_a_		
	Orthostatic hypotension	Yes	41 (39.4%)_a_	49 (37.7%)_a_	30 (38.5%)_a_	0.089	0.977
		No	63 (60.6%)_a_	81 (62.3%)_a_	48 (61.5%)_a_		
	Erectile dysfunction	Yes	40 (38.5%)_a_	48 (36.9%)_a_	26 (33.3%)_a_	0.521	0.791
		No	64 (61.5%)_a_	82 (63.1%)_a_	52 (66.7%)_a_		
Risk factors	First-degree relative with PD	Yes	87 (83.7%)_a,b_	121 (93.1%)_b_	62 (79.5%)_a_	9.239	0.010*
		No	17_a_, (16.3%)_b_	9 (6.9%)_b_	16 (20.5%)_a_		
	Occupational solvent exposure	Yes	56 (53.8%)_a_	83 (63.8%)_a_	51 (65.4%)_a_	3.258	0.192
		No	48 (46.2%)_a_	47 (36.2%)_a_	27 (34.6%)_a_		
	Diabetes mellitus (type II)	Yes	51 (49.0%)_a_	81 (62.3%)_a_	42 (53.8%)_a_	4.281	0.112
		No	53 (51.0%)_a_	49 (37.7%)_a_	36 (46.2%)_a_		
	Regular pesticide exposure	Yes	45 (43.3%)_a_	76 (58.5%)_a_	44 (56.4%)_a_	5.838	0.054
		No	59 (56.7%)_a_	54 (41.5%)_a_	34 (43.6%)_a_		
	Male sex	Yes	49 (47.1%)_a_	71 (54.6%)_a_	41 (52.6%)_a_	1.342	0.515
		No	55 (52.9%)_a_	59 (45.4%)_a_	37 (47.4%)_a_		
	Physical inactivity	Yes	48 (46.2%)_a_	71 (54.6%)_a_	37 (47.4%)_a_	1.927	0.379
		No	56 (53.8%)_a_	59 (45.4%)_a_	41 (52.6%)_a_		
	Intake of tea^#^	Yes	23 (22.1%)_a_	34 (26.2%)_a_	14 (17.9%)_a_	1.854	0.395
		No	81 (77.9%)_a_	96 (73.8%)_a_	64 (82.1%)_a_		
	Intake of caffeine^#^	Yes	21 (20.2%)_a_	15 (11.5%)_a_	8 (10.3%)_a_	4.528	0.109
		No	83 (79.8%)_a_	115 (88.5%)_a_	70 (89.7%)_a_		
	Smoking^#^	Yes	2 (1.9%)_a_	2 (1.5%)_a_	5 (6.4%)_a_	3.915	0.142
		No	102 (98.1%)_a_	128 (98.5%)_a_	73 (93.6%)_a_		
	Low plasma urate levels	Yes	1 (1.0%)_a_	2 (1.5%)_a_	2 (2.6%)_a_	0.917	0.732
		No	103 (99.0%)_a_	128 (98.5%)_a_	76 (97.4%)_a_		
Antiparkinsonian medications	Levodopa	Yes	86 (82.7%)_a,b_	109 (83.8%)_b_	53 (67.9%)_a_	7.973	0.019*
		No	18_a_, (17.3%)_b_	21 (16.2%)_b_	25 (32.1%)_a_		
	Dopamine releasers	Yes	78 (75.0%)_a_	104 (80.0%)_a_	57 (73.1%)_a_	1.583	0.462
		No	26 (25.0%)_a_	26 (20.0%)_a_	21 (26.9%)_a_		
	Anticholinergics	Yes	78 (75.0%)_a_	93 (71.5%)_a,b_	45 (57.7%)_b_	6.608	0.038*
		No	26 (25.0%)_a_	37 (28.5%)_a,b_	33 (42.3%)_b_		
	Dopamine agonists	Yes	67 (64.4%)_a,b_	95 (73.1%)_b_	43 (55.1%)_a_	7.051	0.029*
		No	37_a_, (35.6%)_b_	35 (26.9%)_b_	35 (44.9%)_a_		
	Levodopa decarboxylase inhibitors	Yes	59 (56.7%)_a_	82 (63.1%)_a_	38 (48.7%)_a_	4.121	0.131
		No	45 (43.3%)_a_	48 (36.9%)_a_	40 (51.3%)_a_		
	MAO-B inhibitors	Yes	48 (46.2%)_a_	73 (56.2%)_a_	37 (47.4%)_a_	2.734	0.253
		No	56 (53.8%)_a_	57 (43.8%)_a_	41 (52.6%)_a_		
	COMT inhibitors	Yes	48 (46.2%)_a_	67 (51.5%)_a_	33 (42.3%)_a_	1.762	0.413
		No	56 (53.8%)_a_	63 (48.5%)_a_	45 (57.7%)_a_		

Each subscript letter denotes a subset of professional titles whose column proportions do not differ significantly from each other at the 0.05 level. ^#^Protective factor for PD. ^*^Significant difference at 0.05 level. COMT, catechol-O-methyltransferase; MAO-B, monoamine oxidase-B; PD, Parkinson’s disease.

**Table 4:** Comparisons among categories of hospitals.

Item		Response	Category of hospitals			Statistical comparisons	
			Tertiary hospitals (*n* = 232)	Secondary hospitals (*n* = 35)	Primary hospitals (*n* = 45)	Value	*p*
Motor symptom	Tremor	Yes	221 (95.3%)_a_	33 (94.3%)_a_	43 (95.6%)_a_	0.32	0.906
		No	11 (4.7%)_a_	2 (5.7%)_a_	2 (4.4%)_a_		
	Postural instability	Yes	207 (89.2%)_a_	32 (91.4%)_a_	41 (91.1%)_a_	0.13	1
		No	25 (10.8%)	3 (8.6%)	4 (8.9%)		
	Rigidity	Yes	208 (89.7%)_a_	28 (80.0%)_a_	39 (86.7%)_a_	2.972	0.23
		No	24 (10.3%)_a_	7 (20.0%)_a_	6 (13.3%)_a_		
	Bradykinesia	Yes	177 (76.3%)_a_	30 (85.7%)_a_	33 (73.3%)_a_	1.859	0.393
		No	55 (23.7%)_a_	5 (14.3%)_a_	12 (26.7%)_a_		
Non-motor symptoms	Global cognitive deficit	Yes	169 (72.8%)_a_	24 (68.6%)_a_	32 (71.1%)_a_	0.404	0.815
		No	63 (27.2%)_a_	11 (31.4%)_a_	13 (28.9%)_a_		
	Constipation	Yes	143 (61.6%)_a_	22 (62.9%)_a_	29 (64.4%)_a_	0.136	0.962
		No	89 (38.4%)_a_	13 (37.1%)_a_	16 (35.6%)_a_		
	Sialorrhea	Yes	134 (57.8%)_a_	23 (65.7%)_a_	28 (62.2%)_a_	0.941	0.621
		No	98 (42.2%)_a_	12 (34.3%)_a_	17 (37.8%)_a_		
	Depression (± anxiety)	Yes	129 (55.6%)_a_	20 (57.1%)_a_	27 (60.0%)_a_	0.309	0.878
		No	103 (44.4%)_a_	15 (42.9%)_a_	18 (40.0%)_a_		
	Fatigue	Yes	129 (55.6%)_a_	21 (60.0%)_a_	23 (51.1%)_a_	0.648	0.728
		No	103 (44.4%)_a_	14 (40.0%)_a_	22 (48.9%)_a_		
	Insomnia	Yes	117 (50.4%)_a_	28 (80.0%)_b_	27 (60.0%)_a,b_	11.616	0.003*
		No	115 (49.6%)_a_	7 (20.0%)_b_	18 (40.0%)_a,b_		
	Erectile dysfunction	Yes	115 (49.6%)_a_	17 (48.6%)_a_	22 (48.9%)_a_	0.042	1
		No	117 (50.4%)_a_	18 (51.4%)_a_	23 (51.1%)_a_		
	Urinary dysfunction	Yes	104 (44.8%)_a_	23 (65.7%)_a_	24 (53.3%)_a_	5.795	0.056
		No	128 (55.2%)_a_	12 (34.3%)_a_	21 (46.7%)_a_		
	Restless legs	Yes	106 (45.7%)_a_	20 (57.1%)_a_	20 (44.4%)_a_	1.715	0.436
		No	126 (54.3%)_a_	15 (42.9%)_a_	25 (55.6%)_a_		
	Olfactory loss	Yes	103 (44.4%)_a_	20 (57.1%)_a_	18 (40.0%)_a_	2.541	0.271
		No	129 (55.6%)_a_	15 (42.9%)_a_	27 (60.0%)_a_		
	Orthostatic hypotension	Yes	87 (37.5%)_a_	13 (37.1%)_a_	19 (42.2%)_a_	0.413	0.855
		No	145 (62.5%)_a_	22 (62.9%)_a_	26 (57.8%)_a_		
	Lower back pain	Yes	82 (35.3%)_a_	14 (40.0%)_a_	16 (35.6%)_a_	0.344	0.87
		No	150 (64.7%)_a_	21 (60.0%)_a_	29 (64.4%)_a_		
	Rapid eye movement behavior disorder	Yes	83 (35.8%)_a_	13 (37.1%)_a_	16 (35.6%)_a_	0.065	0.98
		No	149 (64.2%)_a_	22 (62.9%)_a_	29 (64.4%)_a_		
	Diaphoresis (Excessive sweating)	Yes	68 (29.3%)_a_	13 (37.1%)_a_	13 (28.9%)_a_	0.988	0.62
		No	164 (70.7%)_a_	22 (62.9%)_a_	32 (71.1%)_a_		
Prodromal symptoms	Subthreshold parkinsonism or abnormal quantitative motor testing	Yes	152 (65.5%)_a_	23 (65.7%)_a_	34 (75.6%)_a_	1.718	0.461
		No	80 (34.5%)_a_	12 (34.3%)_a_	11 (24.4%)_a_		
	Excessive daytime somnolence	Yes	106 (45.7%)_a_	19 (54.3%)_a_	27 (60.0%)_a_	3.564	0.166
		No	126 (54.3%)_a_	16 (45.7%)_a_	18 (40.0%)_a_		
	Depression (± anxiety)	Yes	103 (44.4%)_a_	20 (57.1%)_a_	25 (55.6%)_a_	3.361	0.185
		No	129 (55.6%)_a_	15 (42.9%)_a_	20 (44.4%)_a_		
	Olfactory loss	Yes	97 (41.8%)_a_	15 (42.9%)_a_	21 (46.7%)_a_	0.402	0.844
		No	135 (58.2%)_a_	20 (57.1%)_a_	24 (53.3%)_a_		
	Urinary dysfunction	Yes	96 (41.4%)_a_	17 (48.6%)_a_	18 (40.0%)_a_	0.757	0.711
		No	136 (58.6%)_a_	18 (51.4%)_a_	27 (60.0%)_a_		
	Rapid eye movement behavior disorder	Yes	92 (39.7%)_a_	16 (45.7%)_a_	20 (44.4%)_a_	0.772	0.697
		No	140 (60.3%)_a_	19 (54.3%)_a_	25 (55.6%)_a_		
	Constipation	Yes	97 (41.8%)_a_	15 (42.9%)_a_	15 (33.3%)_a_	1.189	0.575
		No	135 (58.2%)_a_	20 (57.1%)_a_	30 (66.7%)_a_		
	Orthostatic hypotension	Yes	86 (37.1%)_a_	15 (42.9%)_a_	19 (42.2%)_a_	0.816	0.692
		No	146 (62.9%)_a_	20 (57.1%)_a_	26 (57.8%)_a_		
	Erectile dysfunction	Yes	83 (35.8%)_a_	12 (34.3%)_a_	19 (42.2%)_a_	0.792	0.687
		No	149 (64.2%)_a_	23 (65.7%)_a_	26 (57.8%)_a_		
Risk factors	First-degree relative with PD	Yes	203 (87.5%)_a_	30 (85.7%)_a_	37 (82.2%)_a_	1.143	0.552
		No	29 (12.5%)_a_	5 (14.3%)_a_	8 (17.8%)_a_		
	Occupational solvent exposure	Yes	137 (59.1%)_a_	24 (68.6%)_a_	29 (64.4%)_a_	1.369	0.528
		No	95 (40.9%)_a_	11 (31.4%)_a_	16 (35.6%)_a_		
	Diabetes mellitus (type II)	Yes	124 (53.4%)_a_	26 (74.3%)_a_	24 (53.3%)_a_	5.552	0.062
		No	108 (46.6%)_a_	9 (25.7%)_a_	21 (46.7%)_a_		
	Regular pesticide exposure	Yes	116 (50.0%)_a_	21 (60.0%)_a_	28 (62.2%)_a_	3.022	0.21
		No	116 (50.0%)_a_	14 (40.0%)_a_	17 (37.8%)_a_		
	Male sex	Yes	116 (50.0%)_a_	19 (54.3%)_a_	26 (57.8%)_a_	1.028	0.619
		No	116 (50.0%)_a_	16 (45.7%)_a_	19 (42.2%)_a_		
	Physical inactivity	Yes	115 (49.6%)_a_	19 (54.3%)_a_	22 (48.9%)_a_	0.315	0.896
		No	117 (50.4%)_a_	16 (45.7%)_a_	23 (51.1%)_a_		
	Intake of tea^#^	Yes	49 (21.1%)_a_	9 (25.7%)_a_	13 (28.9%)_a_	1.659	0.433
		No	183 (78.9%)_a_	26 (74.3%)_a_	32 (71.1%)_a_		
	Intake of caffeine^#^	Yes	29 (12.5%)_a_	6 (17.1%)_a_	9 (20.0%)_a_	2.314	0.319
		No	203 (87.5%)_a_	29 (82.9%)_a_	36 (80.0%)_a_		
	Smoking^#^	Yes	5 (2.2%)_a_	1 (2.9%)_a_	3 (6.7%)_a_	2.909	0.148
		No	227 (97.8%)_a_	34 (97.1%)_a_	42 (93.3%)_a_		
	Low plasma urate levels	Yes	3 (1.3%)_a_	0 (0.0%)_a_	2 (4.4%)_a_	2.482	0.245
		No	229 (98.7%)_a_	35 (100.0%)_a_	43 (95.6%)_a_		
Antiparkinsonian medications	Levodopa	Yes	188 (81.0%)_a_	23 (65.7%)_a_	37 (82.2%)_a_	4.331	0.113
		No	44 (19.0%)_a_	12 (34.3%)_a_	8 (17.8%)_a_		
	Dopamine releasers	Yes	176 (75.9%)_a_	26 (74.3%)_a_	37 (82.2%)_a_	0.941	0.631
		No	56 (24.1%)_a_	9 (25.7%)_a_	8 (17.8%)_a_		
	Anticholinergics	Yes	159 (68.5%)_a_	25 (71.4%)_a_	32 (71.1%)_a_	0.175	0.937
		No	73 (31.5%)_a_	10 (28.6%)_a_	13 (28.9%)_a_		
	Dopamine agonists	Yes	152 (65.5%)_a_	22 (62.9%)_a_	31 (68.9%)_a_	0.351	0.85
		No	80 (34.5%)_a_	13 (37.1%)_a_	14 (31.1%)_a_		
	Levodopa decarboxylase inhibitors	Yes	135 (58.2%)_a_	17 (48.6%)_a_	27 (60.0%)_a_	1.31	0.508
		No	97 (41.8%)_a_	18 (51.4%)_a_	18 (40.0%)_a_		
	MAO-B inhibitors	Yes	114 (49.1%)_a_	17 (48.6%)_a_	27 (60.0%)_a_	1.844	0.405
		No	118 (50.9%)_a_	18 (51.4%)_a_	18 (40.0%)_a_		
	COMT inhibitors	Yes	109 (47.0%)_a_	16 (45.7%)_a_	23 (51.1%)_a_	0.327	0.879
		No	123 (53.0%)_a_	19 (54.3%)_a_	22 (48.9%)_a_		

Each subscript letter denotes a subset of categories of hospitals whose column proportions do not differ significantly from each other at the 0.05 level. ^#^Protective factor for PD. ^*^Significant difference at 0.05 level. COMT, catechol-O-methyltransferase; MAO-B, monoamine oxidase-B; PD, Parkinson’s disease.

**Table 5:** Comparisons among education subjects.

Item		Response	Medicine education subjects			Statistical comparisons	
			Chinese medicine (*n* = 130)	Conventional medicine (*n* = 80)	Integrated medicine (*n* = 102)	Value	*p*
Motor symptom	Tremor	Yes	123 (94.6%)_a_	75 (93.8%)_a_	99 (97.1%)_a_	1.305	0.564
		No	7 (5.4%)_a_	5 (6.3%)_a_	3 (2.9%)_a_		
	Postural instability	Yes	117 (90.0%)_a_	71 (88.8%)_a_	92 (90.2%)_a_	0.178	0.943
		No	13 (10.0%)	9 (11.3%)	10 (9.8%)		
	Rigidity	Yes	101 (77.7%)_a_	60 (75.0%)_a_	79 (77.5%)_a_	0.258	0.899
		No	29 (22.3%)_a_	20 (25.0%)_a_	23 (22.5%)_a_		
	Bradykinesia	Yes	117 (90.0%)_a_	71 (88.8%)_a_	87 (85.3%)_a_	1.247	0.525
		No	13 (10.0%)_a_	9 (11.3%)_a_	15 (14.7%)_a_		
Non-motor symptoms	Global cognitive deficit	Yes	95 (73.1%)_a_	57 (71.3%)_a_	73 (71.6%)_a_	0.131	0.96
		No	35 (26.9%)_a_	23 (28.8%)_a_	29 (28.4%)_a_		
	Constipation	Yes	83 (63.8%)_a_	52 (65.0%)_a_	59 (57.8%)_a_	1.232	0.555
		No	47 (36.2%)_a_	28 (35.0%)_a_	43 (42.2%)_a_		
	Sialorrhea	Yes	78 (60.0%)_a_	49 (61.3%)_a_	58 (56.9%)_a_	0.411	0.845
		No	52 (40.0%)_a_	31 (38.8%)_a_	44 (43.1%)_a_		
	Depression (± anxiety)	Yes	76 (58.5%)_a_	51 (63.8%)_a_	49 (48.0%)_a_	4.839	0.087
		No	54 (41.5%)_a_	29 (36.3%)_a_	53 (52.0%)_a_		
	Fatigue	Yes	77 (59.2%)_a_	45 (56.3%)_a_	51 (50.0%)_a_	1.996	0.372
		No	53 (40.8%)_a_	35 (43.8%)_a_	51 (50.0%)_a_		
	Insomnia	Yes	74 (56.9%)_a_	46 (57.5%)_a_	52 (51.0%)_a_	1.061	0.604
		No	56 (43.1%)_a_	34 (42.5%)_a_	50 (49.0%)_a_		
	Erectile dysfunction	Yes	71 (54.6%)_a_	38 (47.5%)_a_	45 (44.1%)_a_	2.662	0.269
		No	59 (45.4%)_a_	42 (52.5%)_a_	57 (55.9%)_a_		
	Urinary dysfunction	Yes	63 (48.5%)_a_	43 (53.8%)_a_	45 (44.1%)_a_	1.664	0.442
		No	67 (51.5%)_a_	37 (46.3%)_a_	57 (55.9%)_a_		
	Restless legs	Yes	59 (45.4%)_a_	38 (47.5%)_a_	49 (48.0%)_a_	0.197	0.907
		No	71 (54.6%)_a_	42 (52.5%)_a_	53 (52.0%)_a_		
	Olfactory loss	Yes	63 (48.5%)_a_	39 (48.8%)_a_	39 (38.2%)_a_	2.967	0.227
		No	67 (51.5%)_a_	41 (51.3%)_a_	63 (61.8%)_a_		
	Orthostatic hypotension	Yes	52 (40.0%)_a_	24 (30.0%)_a_	43 (42.2%)_a_	3.15	0.204
		No	78 (60.0%)_a_	56 (70.0%)_a_	59 (57.8%)_a_		
	Lower back pain	Yes	54 (41.5%)_a_	26 (32.5%)_a_	32 (31.4%)_a_	3.05	0.218
		No	76 (58.5%)_a_	54 (67.5%)_a_	70 (68.6%)_a_		
	Rapid eye movement behavior disorder	Yes	51 (39.2%)_a_	23 (28.8%)_a_	38 (37.3%)_a_	2.489	0.289
		No	79 (60.8%)_a_	57 (71.3%)_a_	64 (62.7%)_a_		
	Diaphoresis (Excessive sweating)	Yes	42 (32.3%)_a_	24 (30.0%)_a_	28 (27.5%)_a_	0.641	0.744
		No	88 (67.7%)_a_	56 (70.0%)_a_	74 (72.5%)_a_		
Prodromal symptoms	Subthreshold parkinsonism or abnormal quantitative motor testing	Yes	89 (68.5%)_a_	52 (65.0%)_a_	68 (66.7%)_a_	0.3	0.853
		No	41 (31.5%)_a_	28 (35.0%)_a_	34 (33.3%)_a_		
	Excessive daytime somnolence	Yes	68 (52.3%)_a_	37 (46.3%)_a_	47 (46.1%)_a_	1.152	0.569
		No	62 (47.7%)_a_	43 (53.8%)_a_	55 (53.9%)_a_		
	Depression (± anxiety)	Yes	68 (52.3%)_a_	35 (43.8%)_a_	45 (44.1%)_a_	2.114	0.343
		No	62 (47.7%)_a_	45 (56.3%)_a_	57 (55.9%)_a_		
	Olfactory loss	Yes	57 (43.8%)_a_	34 (42.5%)_a_	42 (41.2%)_a_	0.177	0.905
		No	73 (56.2%)_a_	46 (57.5%)_a_	60 (58.8%)_a_		
	Urinary dysfunction	Yes	60 (46.2%)_a_	28 (35.0%)_a_	43 (42.2%)_a_	2.522	0.28
		No	70 (53.8%)_a_	52 (65.0%)_a_	59 (57.8%)_a_		
	Rapid eye movement behavior disorder	Yes	57 (43.8%)_a_	28 (35.0%)_a_	43 (42.2%)_a_	1.679	0.436
		No	73 (56.2%)_a_	52 (65.0%)_a_	59 (57.8%)_a_		
	Constipation	Yes	61 (46.9%)_a_	23 (28.8%)_b_	43 (42.2%)_a,b_	6.981	0.029*
		No	69 (53.1%)_a_	57 (71.3%)_b_	59 (57.8%)_a,b_		
	Orthostatic hypotension	Yes	53 (40.8%)_a_	26 (32.5)_a_	41 (40.2%)_a_	1.622	0.448
		No	77 (59.2%)_a_	54 (67.5%)_a_	61 (59.8%)_a_		
	Erectile dysfunction	Yes	48 (36.9%)_a_	32 (40.0%)_a_	34 (33.3%)_a_	0.886	0.634
		No	82 (63.1%)_a_	48 (60.0%)_a_	68 (66.7%)_a_		
Risk factors	First-degree relative with PD	Yes	115 (88.5%)_a_	68 (85.0%)_a_	87 (85.3%)_a_	0.785	0.676
		No	15 (11.5%)_a_	12 (15.0%)_a_	15 (14.7%)_a_		
	Occupational solvent exposure	Yes	76 (58.5%)_a_	53 (66.3%)_a_	61 (59.8%)_a_	1.334	0.522
		No	54 (41.5%)_a_	27 (33.8%)_a_	41 (40.2%)_a_		
	Diabetes mellitus (type II)	Yes	79 (60.8%)_a_	49 (61.3%)_a_	46 (45.1%)_b_	6.927	0.032*
		No	51 (39.2%)_a_	31 (38.8%)_a_	56 (54.9%)_b_		
	Regular pesticide exposure	Yes	71 (54.6%)_a_	43 (53.8%)_a_	51 (50.0%)_a_	0.53	0.787
		No	59 (45.4%)_a_	37 (46.3%)_a_	51 (50.0%)_a_		
	Male sex	Yes	71 (54.6%)_a_	44 (55.0%)_a_	46 (45.1%)_a_	2.56	0.283
		No	59 (45.4%)_a_	36 (45.0%)_a_	56 (54.9%)_a_		
	Physical inactivity	Yes	60 (46.2%)_a_	43 (53.8%)_a_	53 (52.0%)_a_	1.378	0.516
		No	70 (53.8%)_a_	37 (46.3%)_a_	49 (48.0%)_a_		
	Intake of tea^#^	Yes	28 (21.5%)_a_	24 (30.0%)_a_	19 (18.6%)_a_	3.395	0.179
		No	102 (78.5%)_a_	56 (70.0%)_a_	83 (81.4%)_a_		
	Intake of caffeine^#^	Yes	14 (10.8%)_a_	15 (18.8%)_a_	15 (14.7%)_a_	2.69	0.255
		No	116 (89.2%)_a_	65 (81.3%)_a_	87 (85.3%)_a_		
	Smoking^#^	Yes	4 (3.1%)_a_	2 (2.5%)_a_	3 (2.9%)_a_	0.166	1
		No	126 (96.9%)_a_	78 (97.5%)_a_	99 (97.1%)_a_		
	Low plasma urate levels	Yes	4 (3.1%)_a_	0 (0.0%)_a_	1 (1.0%)_a_	2.542	0.318
		No	126 (96.9%)_a_	80 (100.0%)_a_	101 (99.0%)_a_		
Antiparkinsonian medications	Levodopa	Yes	104 (80.0%)_a_	65 (81.3%)_a_	79 (77.5%)_a_	0.435	0.82
		No	26 (20.0%)_a_	15 (18.8%)_a_	23 (22.5%)_a_		
	Dopamine releasers	Yes	93 (71.5%)_a_	66 (82.5%)_a_	80 (78.4%)_a_	3.506	0.172
		No	37 (28.5%)_a_	14 (17.5%)_a_	22 (21.6%)_a_		
	Anticholinergics	Yes	82 (63.1%)_a_	61 (76.3%)_a_	73 (71.6%)_a_	4.343	0.111
		No	48 (36.9%)_a_	19 (23.8%)_a_	29 (28.4%)_a_		
	Dopamine agonists	Yes	86 (66.2%)_a_	52 (65.0%)_a_	67 (65.7%)_a_	0.048	0.988
		No	44 (33.8%)_a_	28 (35.0%)_a_	35 (34.3%)_a_		
	Levodopa decarboxylase inhibitors	Yes	71 (54.6%)_a_	52 (65.0%)_a_	56 (54.9%)_a_	2.564	0.276
		No	59 (45.4%)_a_	28 (35.0%)_a_	46 (45.1%)_a_		
	MAO-B inhibitors	Yes	67 (51.5%)_a_	45 (56.3%)_a_	46 (45.1%)_a_	2.295	0.319
		No	63 (48.5%)_a_	35 (43.8%)_a_	56 (54.9%)_a_		
	COMT inhibitors	Yes	61 (46.9%)_a_	39 (48.8%)_a_	48 (47.1%)_a_	0.088	0.968
		No	69 (53.1%)_a_	41 (51.3%)_a_	54 (52.9%)_a_		

Each subscript letter denotes a subset of education subjects whose column proportions do not differ significantly from each other at the 0.05 level. *Significant different at 0.05 level; ^#^Protective factors for PD. COMT, catechol-O-methyltransferase; MAO-B, monoamine oxidase-B; PD, Parkinson’s disease.

Among respondents holding different medical degrees, statistically significant differences were observed only in the recognition of specific risk factors. Notably, those with a master’s degree demonstrated a lower rate of identifying constipation as a risk factor compared to those with a bachelor’s degree (28.8% vs. 46.9%, *p* = 0.029). In addition, respondents with doctoral degrees were less likely to identify diabetes mellitus as a risk factor for PD, compared to those with master’s degrees (45.1% vs. 61.3%) or bachelor’s degrees (45.1% vs. 60.8%) (*p* = 0.032) (Table 2).

In the context of professional titles, chief or associate chief physicians outperformed residents in recognizing bradykinesia (83.7% vs. 66.7%, *p* = 0.028) and anticholinergics (75.0% vs. 57.7%, *p* = 0.038). Attendings demonstrated higher awareness of familial PD risk (93.1% vs. 79.5%, *p* = 0.01) and medications (levodopa: 83.8% vs. 67.9%; dopamine agonists: 73.1% vs. 55.1%, *p* < 0.03) than residents (Table 3).

Respondents from tertiary hospitals recognized insomnia as a non-motor symptom less than secondary hospital peers (50.4% vs. 80%, *p* = 0.003), though no other symptom or medication differences were significant (Table 4).

By education background, respondents majoring conventional medicine recognized constipation as a prodromal symptom less than those studying Chinese medicine (28.8% vs. 46.9%, *p* = 0.029). Interestingly, those pursuing integrated medicine showed significantly reduced awareness of diabetes mellitus as a risk factor for PD, with only 45.1% recognizing it as such, contrasted to respondents majoring in either Chinese medicine (60.8%) or conventional medicine (61.3%) (*p* = 0.032) (Table 5).

#### 3.2.4 Multiple regression analysis between demographic factors and knowledge of PD

Multiple regression analyses were conducted to identify potential relationships between demographic factors and correct response to knowledge of PD. All surveyed items related to PD knowledge, including motor symptoms, non-motor symptoms, prodromal symptoms, risk and protective factors, and antiparkinsonian medications, were initially considered as dependent variables. Regression analyses that were successfully constructed and met the statistical criteria for inclusion can be found in Supplementary file 3.

The results indicate that, as age increases, the physicians seem to be less aware of rigidity as a motor symptom but know more about PD risk factors like the first-degree relative with PD and physical inactivity. Compared to physicians working outside neurology department, those working in neurology departments were more aware of non-motor symptoms like constipation, orthostatic hypotension, urinary dysfunction, olfactory loss, lower back pain, RBD and diaphoresis, prodromal symptom of constipation, protective factors of consumption of tea and caffeine, risk factors of first-degree relative with PD and low plasma urate levels. Categories of hospitals result in differences in knowing insomnia as a non-motor symptom. Professional titles lead to differences in knowing risk factors of first-degree relatives with PD and physical inactivity, antiparkinsonian medication of levodopa and dopamine agonists. In addition, medical practice experience contributes to different knowledge of physical inactivity as a risk factor, while education subjects contribute to differences of knowing diabetes mellitus as a risk factor.

## 4 Discussion

### 4.1 Representativeness of the samples

The cross-sectional survey encompassed 312 respondents working in diverse departments across 64 hospitals of varying levels in 17 cities. These respondents boasted a range of educational qualifications and professional titles, spanning different medical specialities. This diversity in demographic information not only enhances the representativeness of the respondents but also contributes to the increased generalizability of our findings.

However, it is worth noting that approximately three-quarters of the respondents come from tertiary hospitals, whereas the estimated ratio of tertiary hospitals to other hospitals in Guangdong province stands at around 1: 4. Intriguingly, despite this preponderance, we found no statistical differences among hospital categories in relation to the surveyed issues, with the exception of non-motor symptom of insomnia. Therefore, the preponderance of respondents from tertiary hospitals did not significantly hamper the generalizability of our findings to primary and secondary hospitals.

Nevertheless, the fact that respondents primarily from tertiary hospitals, which are expected to demonstrate superior medical knowledge, displayed limited awareness of PD knowledge, underscores a broader issue of low PD knowledge awareness across hospitals in Guangdong Province. This observation is particularly concerning as tertiary hospitals are often considered the leaders in medical research and education. The limited PD knowledge among these respondents suggests that there is a need for more widespread and targeted educational efforts to enhance PD awareness and understanding across all levels of hospitals in Guangdong Province.

### 4.2 Summary of PD knowledge

#### 4.2.1 Motor symptom

Bradykinesia stands as the most defining clinical features of PD (Ling et al., 2012), serving as the cornerstone for diagnosing the condition among its four motor symptoms (Postuma et al., 2015). Nevertheless, it was discovered to the least recognized among these symptoms. Notably, resident physicians demonstrated a significant lack of awareness regarding bradykinesia as a motor symptom of PD, compared to (associate) chief physicians, likely due to their fewer years of clinical practice. This low awareness of bradykinesia aligns with findings from a prior survey in Shanghai, China, which revealed that bradykinesia tend to take longer to be diagnosed compared to tremor (朱莹莹 et al., 2015).

Unsurprisingly, tremor emerged as the most recognized motor symptom, constant with a prior report (Zach et al., 2015). It is estimated that an average of 79.9% of individuals with PD exhibit tremors, yet notably, up to 19.9% do not (Gupta et al., 2020). It is important to highlight that tremor can also manifest in other conditions, such as essential tremor (Pan and Kuo, 2022), thereby emphasizing the significance of a thorough differential diagnosis.

The multiple regression analysis indicated that rigidity was less recognized as a motor symptom among physicians as their age increased. This could be attributed to the fact that rigidity can also arise from normal aging or arthritis (Ertan et al., 1999), leading to confusion in differential diagnosis.

#### 4.2.2 Non-motor symptoms

It has been documented that over 90% of patients with PD manifest at least one non-motor symptom in the early stages (Frucht, 2004), and no PD patient is exempt from these non-motor symptoms throughout the course of their illness (Carroll et al., 2021; Chaudhuri et al., 2021; Gulunay et al., 2020). The high prevalence of non-motor symptoms underscores their significance in the early detection, recognition and diagnosis of PD.

However, our survey indicates that non-PD specialists often fail to adequately recognize these non-motor symptoms. Even the most common non-motor symptom, such as constipation, insomnia, fatigue, drooling, depression and anxiety, are reported to be under-recognized and undertreated by physicians, despite their widespread prevalence (American Parkinson Disease Association, 2023; Garrison et al., 2021; Miller et al., 2019; Shulman et al., 2002). The lack of recognition may stem from the non-specific and inclusive nature of symptoms like excessive sweating and pain, while symptoms like RBD and restless legs are less frequently encountered.

Interestingly, we found that physicians from tertiary hospitals were less aware of insomnia as a non-motor symptom, compared to those from secondary hospitals. This may be attributed to the high level of specialization in tertiary hospitals, which could result in a narrower focus and a lack of knowledge across multiple disciplines (王宇朋, 王萍, 2021; 韩笑, 王瑞, 2022). In addition, it was not surprising that non-PD specialists from neurology department know better about non-motor symptoms when compared to those not working in neurology departments, after taking all demographic information into consideration.

The widespread under-recognition of non-motor symptoms indicates a gap in knowledge about PD among non-PD specialists and highlight the necessity of education and training. Enhancing the awareness and understanding of non-motor symptoms among these specialists is crucial for improving diagnostic accuracy and facilitating timely therapeutic interventions (Shulman et al., 2002).

#### 4.2.3 Prodromal symptoms

Prodromal PD refers to the preclinical stage, during which individuals do not yet fulfill the diagnostic criteria for PD but exhibit signs and symptoms. Prodromal symptoms predict an increased risk of developing motor symptoms and a future diagnosis of PD (Postuma et al., 2015). Notably, most prodromal symptoms are non-motor and significantly impact the quality of life of individuals in both the prodromal stage and those who have progressed to motor-PD. Consequently, the early detection and management of these prodromal symptoms are crucial for providing optimal care (Mantri and Morley, 2018; Plouvier et al., 2014).

It is noteworthy that less than half of non-PD specialists are aware of most prodromal symptoms, excluding subthreshold parkinsonism, despite their high predictive value for developing PD. This includes symptoms such as RBD. The under-awareness of prodromal symptoms may be attributed to their non-specific nature, or relatively low prevalence. Interestingly, surveyed physicians with a master’s degree demonstrated less awareness of constipation as a prodromal symptom compared to those with a bachelor or doctoral degrees. Furthermore, physicians trained in western medicine demonstrated less awareness of constipation as a prodromal symptom than those with an education history in Chinese medicine. However, after controlling all surveyed demographic information, these differences got insignificant.

#### 4.2.4 Risk and protective factors

Modifiable risk factors and protective measures, including tea and coffee consumption, smoking habits, physical activity, exposure to solvents and pesticides, as well as plasma urate levels, can significantly influence the development and progression of PD (Belvisi et al., 2020). Nevertheless, it has been observed that surveyed physicians lack sufficient awareness of these factors. This limited understanding among physicians may stem from insufficient medical education focused on non-PD specialists, potentially contributing to unfavorable outcomes in disease progression.

#### 4.2.5 Antiparkinsonian medications

Twenty percent of the surveyed physicians remain unaware of levodopa as an antiparkinsonian medication, despite being the first-line treatment choice (Health NIf, Excellence C, 2017; Waller et al., 2021). In addition, COMT inhibitors are the least well-known medication, despite having a long history of clinical use since 1998 (First COMT inhibitor approved for Parkinson's disease, 1998). Moreover, several antiparkinsonian medications including levodopa, anticholinergics and dopamine agonists, are less familiar to resident physicians, potentially due to their limited clinical experience. These findings may explain the GPs’ lack of confidence in prescribing antiparkinsonian medications (Lim et al., 2022). Inappropriate utilization of antiparkinsonian medications can lead to motor complications, side effects, and negatively impact the progression of PD (Connolly and Lang, 2014). Therefore, it is imperative to enhance knowledge of PD medications among non-PD specialists.

### 4.3 Implication for clinical practice and research

The survey reveals insufficient knowledge of PD diagnosis and treatment, involving prodroma symptom, non-motor symptoms, risk and protective factors, and antiparkinsonian medications, among non-PD specialists. It has been established that the scarcity of specialized physicians in primary and secondary hospitals is a significant obstacle to early diagnosis of PD (Li et al., 2019). Moreover, education, training and qualifications among physicians in China’s primary care system has also been widely documented (Li et al., 2017). Our results underscore the urgent need for specialized education and training for non-PD specialists to enhance early and timely diagnosis, as well as appropriate management strategies of the disease (徐静, 2015). The effects of specialized education and training in improving diagnostic accuracy and efficiency can be proposed to be evaluated in future research.

### 4.4 Limitations

A significant limitation of this study is its generalizability. Firstly, as the survey was conducted solely in south China, the findings may not be fully applicable to physicians residing in other regions of China. Secondly, given the disparities in healthcare systems between China and other nations, the conclusions drawn from this study may not be directly transferable to non-PD specialists practicing in countries with different healthcare frameworks. Nonetheless, given China’s substantial contribution to the global PD burden and the representativeness of its healthcare challenges among low- and middle-income countries, the findings may still hold relevance for similar contexts. The study’s emphasis on fundamental clinical challenges—such as patient education, multidisciplinary coordination, and resource limitations—may offer valuable perspectives for practitioners in comparable settings. Finally, the convenience sampling method employed in this research may also constrain the generalizability of the results to a broader population. Despite these limitations, the findings provide a foundational understanding of the challenges faced by physicians in managing PD within resource-constrained environments, underscoring the need for context-specific strategies to optimize care delivery.

## 5 Conclusion

The knowledge of PD non-motor symptoms, prodromal symptoms, risk and protective factors, as well as antiparkinsonian medications, was relatively inadequate among non-PD specialists in south China. To improve the quality of healthcare for PD patients in this region, it is imperative to develop targeted education and training strategies aimed at enhancing the knowledge of PD among non-PD specialists.

## Data Availability

The original contributions presented in the study are included in the article/Supplementary material, further inquiries can be directed to the corresponding authors.

## Footnotes

^1^ https://www.wjx.cn/
