# Supplementary material for: Knowledge of Parkinson’s disease among non-PD specialists: a web-based survey in South China
Source: Front Aging Neurosci. 2025 Apr 9;17:1488009. doi: 10.3389/fnagi.2025.1488009 (PMC12014546; doi:10.3389/fnagi.2025.1488009)
Supplement: Supplementary file 1 [file Table_1.DOCX]

Survey about Knowledge of Parkinson's Disease Among Non-Parkinson's Physicians

Dear doctor,

Thank you for participating in this survey!

The survey was designed and implemented by Neurology Department in Guangdong Provincial Hospital of Chinese Medicine. The project has been approved by the Ethics Committee of Guangdong Provincial Hospital of Chinese Medicine (ZM2023-393). The survey aims to know about the general knowledge about symptoms, risk and protective factors, and treatment of Parkinson’s disease among physicians not specializing in Parkinson’s disease. This survey consists of 47 questions about Parkinson’s disease and will take approximately 15 minutes to complete.

Thank you again for your support.

Neurology department, Guangdong Provincial Hospital of Chinese Medicine

**Section 1: Demographic information:**

Question 1: What is your professional title in medicine?

A: Chief Physician; B: Associate Chief Physician; C: Attending Physician; D: Resident Physician; E: Intern physician

Question 2: What is your educational background in medicine?

A: Traditional Chinese Medicine + Western Medicine; B: Integrated Traditional Chinese and Western Medicine; C: Western Medicine; D: Traditional Chinese Medicine; E: Other _________

Question 3: What is your highest degree in medicine?

A: Diploma and Below; B: Bachelor's Degree; C: Master's Degree; D: Doctorate and Above

Question 4: Which Province do you work in?

A. Guangdong Province B. Others _________

Question 5: What is the level of the hospital you work in?

A: Community Hospital (First-Grade A, First-Grade B); B: Second-Tier Hospital (Second-Grade A, Second-Grade B); C: Third-Tier Hospital (Third-Grade A, Third-Grade B, Special Grade); D: Other ___________

Question 6: How many years have you practiced as a physician? ________ Years

Question 7.1: Which specialty did you study when you were a medical student?

A: Neurology; B: Neurosurgery; C: Geriatrics; D: Cardiology; E: Orthopedics; F: Surgery; G: Gynecology; H: Pediatrics; I: Emergency Medicine; J: Critical Care Medicine; K: Gastroenterology; L: Endocrinology; M: Respiratory; N: Nephrology; O: Oncology; P: Psychiatry; Q: Other _________

Question 7.2 If Neurology is selected above:

Does your research during student period involve Parkinson's disease?

A: Yes; B: No; C: Unsure

Question 8.1: Which department(s) have you worked in? (Multiple choices are allowed)

A: Neurology; B: Neurosurgery; C: Geriatrics; D: Cardiology; E: Orthopedics; F: Surgery; G: Gynecology; H: Pediatrics; I: Emergency Medicine; J: Critical Care Medicine; K: Gastroenterology; L: Endocrinology; M: Respiratory; N: Nephrology; O: Oncology; P: Psychiatry; Q: Other _________

Question 8.2: If Neurology is selected above:

Is Parkinson’s disease a specialty in your department?

A: Yes; B: No; C: Unsure

**Section 2: Knowledge about Parkinson’s disease**

Question 9.1: Is tremor a typical manifestation of Parkinson's disease?

A: I do not understand/recognize the symptom; B: I am not sure; C: Yes, it is a motor symptom of PD; D: No, it is not a motor symptom of PD

Question 9.2: Is bradykinesia a typical manifestation of Parkinson's disease?

A: I do not understand/recognize the symptom; B: I am not sure; C: Yes, it is a motor symptom of PD; D: No, it is not a motor symptom of PD

Question 9.3: Is rigidity a typical manifestation of Parkinson's disease?

A: I do not understand/recognize the symptom; B: I am not sure; C: Yes, it is a motor symptom of PD; D: No, it is not a motor symptom of PD

Question 9.4: Is postural instability a typical manifestation of Parkinson's disease?

A: I do not understand/recognize the symptom; B: I am not sure; C: Yes, it is a motor symptom of PD; D: No, it is not a motor symptom of PD

Question 10.1: Is constipation a non-motor symptom of Parkinson’s disease?

A: I do not understand/recognize the symptom; B: I am not sure; C: Yes, it is a non-motor symptom of PD; D: No, it is not a non-motor symptom of PD

Question 10.2: Is orthostatic hypotension a non-motor symptom of Parkinson’s disease?

A: I do not understand/recognize the symptom; B: I am not sure; C: Yes, it is a non-motor symptom of PD; D: No, it is not a non-motor symptom of PD

Question 10.3: Is salivation a non-motor symptom of Parkinson’s disease?

A: I do not understand/recognize the symptom; B: I am not sure; C: Yes, it is a non-motor symptom of PD; D: No, it is not a non-motor symptom of PD

Question 10.4: Is urinary dysfunction a non-motor symptom of Parkinson’s disease?

A: I do not understand/recognize the symptom; B: I am not sure; C: Yes, it is a non-motor symptom of PD; D: No, it is not a non-motor symptom of PD

Question 10.5: Is insomnia a non-motor symptom of Parkinson’s disease?

A: I do not understand/recognize the symptom; B: I am not sure; C: Yes, it is a non-motor symptom of PD; D: No, it is not a non-motor symptom of PD

Question 10.6: Is Olfactory loss a non-motor symptom of Parkinson’s disease?

A: I do not understand/recognize the symptom; B: I am not sure; C: Yes, it is a non-motor symptom of PD; D: No, it is not a non-motor symptom of PD

Question 10.7: Is low Back Pain a non-motor symptom of Parkinson’s disease?

A: I do not understand/recognize the symptom; B: I am not sure; C: Yes, it is a non-motor symptom of PD; D: No, it is not a non-motor symptom of PD

Question 10.8: Is rapid eye movement behavior disorder a non-motor symptom of Parkinson’s disease?

A: I do not understand/recognize the symptom; B: I am not sure; C: Yes, it is a non-motor symptom of PD; D: No, it is not a non-motor symptom of PD

Question 10.9: Is diaphoresis a non-motor symptom of Parkinson’s disease?

A: I do not understand/recognize the symptom; B: I am not sure; C: Yes, it is a non-motor symptom of PD; D: No, it is not a non-motor symptom of PD

Question 10.10: Is anxiety and/or depression a non-motor symptom of Parkinson’s disease?

A: I do not understand/recognize the symptom; B: I am not sure; C: Yes, it is a non-motor symptom of PD; D: No, it is not a non-motor symptom of PD

Question 10.11: Is restless legs syndrome a non-motor symptom of Parkinson’s disease?

A: I do not understand/recognize the symptom; B: I am not sure; C: Yes, it is a non-motor symptom of PD; D: No, it is not a non-motor symptom of PD

Question 10.12: Is global cognitive deficit a non-motor symptom of Parkinson’s disease?

A: I do not understand/recognize the symptom; B: I am not sure; C: Yes, it is a non-motor symptom of PD; D: No, it is not a non-motor symptom of PD

Question 10.13: Is erectile dysfunction a non-motor symptom of Parkinson’s disease?

A: I do not understand/recognize the symptom; B: I am not sure; C: Yes, it is a non-motor symptom of PD; D: No, it is not a non-motor symptom of PD

Question 10.14: Is fatigue a non-motor symptom of Parkinson’s disease?

A: I do not understand/recognize the symptom; B: I am not sure; C: Yes, it is a non-motor symptom of PD; D: No, it is not a non-motor symptom of PD

Question 11.1: Is subthreshold parkinsonism or abnormal quantitative motor testing a prodromal symptom of Parkinson’s disease?

A: I do not understand/recognize the symptom; B: I am not sure; C: Yes, it is a prodromal symptom of PD; D: No, it is not a prodromal symptom of PD

Question 11.2: Is excessive daytime somnolence a prodromal symptom of Parkinson’s disease?

A: I do not understand/recognize the symptom; B: I am not sure; C: Yes, it is a prodromal symptom of PD; D: No, it is not a prodromal symptom of PD

Question 11.3: Is depression and/or anxiety a prodromal symptom of Parkinson’s disease?

A: I do not understand/recognize the symptom; B: I am not sure; C: Yes, it is a prodromal symptom of PD; D: No, it is not a prodromal symptom of PD

Question 11.4: Is olfactory loss a prodromal symptom of Parkinson’s disease?

A: I do not understand/recognize the symptom; B: I am not sure; C: Yes, it is a prodromal symptom of PD; D: No, it is not a prodromal symptom of PD

Question 11.5: Is urinary dysfunction a prodromal symptom of Parkinson’s disease?

A: I do not understand/recognize the symptom; B: I am not sure; C: Yes, it is a prodromal symptom of PD; D: No, it is not a prodromal symptom of PD

Question 11.6: Is rapid eye movement behavior disorder a prodromal symptom of Parkinson’s disease?

A: I do not understand/recognize the symptom; B: I am not sure; C: Yes, it is a prodromal symptom of PD; D: No, it is not a prodromal symptom of PD

Question 11.7: Is constipation a prodromal symptom of Parkinson’s disease?

A: I do not understand/recognize the symptom; B: I am not sure; C: Yes, it is a prodromal symptom of PD; D: No, it is not a prodromal symptom of PD

Question 11.8: Is orthostatic hypotension a prodromal symptom of Parkinson’s disease?

A: I do not understand/recognize the symptom; B: I am not sure; C: Yes, it is a prodromal symptom of PD; D: No, it is not a prodromal symptom of PD

Question 11.9: Is erectile dysfunction a prodromal symptom of Parkinson’s disease?

A: I do not understand/recognize the symptom; B: I am not sure; C: Yes, it is a prodromal symptom of PD; D: No, it is not a prodromal symptom of PD

Question 12.1 Is male gender a risk or protective factor for Parkinson’s disease?

A: I do not understand the factor; B: I am not sure; C: It is a risk factor for PD; D: It is a protective factor for PD

Question 12.2 Is having a first-degree relative with PD a risk or protective factor for Parkinson’s disease?

A: I do not understand the factor; B: I am not sure; C: It is a risk factor for PD; D: It is a protective factor for PD

Question 12.3 Is occupational solvent exposure a risk or protective factor for Parkinson’s disease?

A: I do not understand the factor; B: I am not sure; C: It is a risk factor for PD; D: It is a protective factor for PD

Question 12.4 Is diabetes mellitus (type II) a risk or protective factor for Parkinson’s disease?

A: I do not understand the factor; B: I am not sure; C: It is a risk factor for PD; D: It is a protective factor for PD

Question 12.5 Is regular pesticide exposure a risk or protective factor for Parkinson’s disease?

A: I do not understand the factor; B: I am not sure; C: It is a risk factor for PD; D: It is a protective factor for PD

Question 12.6 Is physical inactivity a risk or protective factor for Parkinson’s disease?

A: I do not understand the factor; B: I am not sure; C: It is a risk factor for PD; D: It is a protective factor for PD

Question 12.7 Is intake of tea a risk or protective factor for Parkinson’s disease?

A: I do not understand the factor; B: I am not sure; C: It is a risk factor for PD; D: It is a protective factor for PD

Question 12.8 Is intake of caffeine a risk or protective factor for Parkinson’s disease?

A: I do not understand the factor; B: I am not sure; C: It is a risk factor for PD; D: It is a protective factor for PD

Question 12.9 Is smoking a risk or protective factor for Parkinson’s disease?

A: I do not understand the factor; B: I am not sure; C: It is a risk factor for PD; D: It is a protective factor for PD

Question 12.10 Is low plasma urate levels a risk or protective factor for Parkinson’s disease?

A: I do not understand the factor; B: I am not sure; C: It is a risk factor for PD; D: It is a protective factor for PD

Question 13.1 Do anticholinergic drugs (e.g., trihexyphenidyl, etc.) belong to antiparkinsonian medication?

A: I don’t know; B: Yes, they do; C: No, they do not

Question 13.2 Do dopamine releasers (e.g., amantadine, etc.) belong to antiparkinsonian medication?

A: I don’t know; B: Yes, they do; C: No, they do not

Question 13.3 Do dopamine replacement drugs (e.g., levodopa, carbidopa-levodopa combinations, etc.) belong to antiparkinsonian medication?

A: I don’t know; B: Yes, they do; C: No, they do not

Question 13.4 Do dopamine receptor agonists (e.g., pramipexole, rotigotine, piribedil, ropinirole, etc.) belong to antiparkinsonian medication?

A: I don’t know; B: Yes, they do; C: No, they do not

Question 13.5 Do MAO-B inhibitors (e.g., selegiline, rasagiline, and safinamide, etc.) belong to antiparkinsonian medication?

A: I don’t know; B: Yes, they do; C: No, they do not

Question 13.6 Do COMT inhibitors (e.g., entacapone and opicapone, etc.) belong to antiparkinsonian medication?

A: I don’t know; B: Yes, they do; C: No, they do not

Question 13.7 Do dual inhibitors of levodopa metabolism (e.g., Madopar HBS, etc.) belong to antiparkinsonian medication?

A: I don’t know; B: Yes, they do; C: No, they do not
