# Supplementary material for: Knowledge of Parkinson’s disease among non-PD specialists: a web-based survey in South China
Source: Front Aging Neurosci. 2025 Apr 9;17:1488009. doi: 10.3389/fnagi.2025.1488009 (PMC12014546; doi:10.3389/fnagi.2025.1488009)
Supplement: Supplementary file 2 [file Table_2.DOCX]

Supplementary file 2 Characteristics of respondents

| **Characteristics** | | **Mean (SD)** |
| --- | --- | --- |
| Age (years) | | 37.59 (6.82) |
| Medical practice experience (years) | | 12.04 (7.74) |
| **Categories** | | **Number (%)** |
| Categories of hospital | Tertiary hospitals | 232 (74.36) |
|  | Secondary hospitals | 35 (11.22) |
|  | Primary hospitals | 45 (14.42) |
| Departments | Neurology department | 21 (6.73) |
|  | Non-neurology department | 291 (93.27) |
| Education qualification | Bachelor’ degree in medicine or below | 125 (40.06) |
|  | Master’s degree in medicine | 164 (52.56) |
|  | Doctoral degree in medicine | 23 (7.37) |
| Education subject | Chinese medicine | 130 (41.67) |
|  | Conventional medicine | 80 (25.64) |
|  | Integrated Chinese and conventional medicine | 102 (32.69) |
| Professional titles | Associate chief physician or Chief Physician | 104 (33.33) |
|  | Attending physician | 130 (41.67) |
|  | Resident physician | 78 (25.00) |

Note: SD: standardized deviation
