# Supplementary material for: Knowledge of Parkinson’s disease among non-PD specialists: a web-based survey in South China
Source: Front Aging Neurosci. 2025 Apr 9;17:1488009. doi: 10.3389/fnagi.2025.1488009 (PMC12014546; doi:10.3389/fnagi.2025.1488009)
Supplement: Supplementary file 3 [file Table_3.DOCX]

Supplementary file 3 Regression analysis of contributing factors to differences of knowledge in Parkinson’s disease

| Dimension | Dependent variables (correct response to PD knowledge) | Independent variables | *p* | Exp(B) | 95% CI | | |
| --- | --- | --- | --- | --- | --- | --- | --- |
|  |  |  |  |  | Lower | Upper | |
| Motor symptoms | Rigidity | Age (years) | 0.003 | 0.936 | 0.896 | 0.978 | |
| Non-motor symptoms | Constipation | Departments (Neurology compared to non-neurology) | 0.015 | 6.297 | 1.440 | 27.547 | |
|  | Orthostatic hypotension | Departments (Neurology compared to non-neurology) | 0.008 | 3.543 | 1.386 | 9.054 | |
|  | Urinary dysfunction | Departments (Neurology compared to non-neurology) | 0.035 | 2.849 | 1.075 | 7.549 | |
|  | Insomnia | Categories of hospitals | 0.006 |  |  |  | |
|  |  | Tertiary hospitals compared to primary hospitals | 0.241 | 1.474 | 0.770 | 2.823 | |
|  |  | Secondary hospitals compared to primary hospitals | 0.060 | 0.375 | 0.135 | 1.041 | |
|  | Olfactory loss | Departments (Neurology compared to non-neurology) | 0.006 | 4.250 | 1.516 | 11.911 | |
|  | Lower back pain | Departments (Neurology compared to non-neurology) | 0.014 | 3.152 | 1.264 | 7.857 | |
|  | RBD | Departments (Neurology compared to non-neurology) | 0.041 | 2.547 | 1.038 | 6.248 | |
|  | Diaphoresis (Excessive sweating) | Departments (Neurology compared to non-neurology) | 0.008 | 3.398 | 1.380 | 8.369 | |
| Prodromal symptoms | Constipation | Departments (Neurology compared to non-neurology) | 0.008 | 3.398 | 1.380 | 8.369 | |
| Risk and protective factors | Intake of Tea | Departments (Neurology compared to non-neurology) | 0.002 | 4.235 | 1.718 | 10.440 | |
|  | Intake of caffeine | Departments (Neurology compared to non-neurology) | 0.013 | 3.432 | 1.300 | 9.060 | |
|  | First-degree relative with PD | Age (years) | 0.005 | 1.097 | 1.029 | 1.170 | |
|  |  | Professional titles | 0.001 |  |  |  | |
|  |  | Professional titles (Chief physicians compared to resident physicians) | 0.013 | 0.221 | 0.067 | 0.731 | |
|  |  | Professional titles (Attending physicians compared to resident physicians) | 0.000 | 0.151 | 0.054 | 0.419 | |
|  |  | Departments (Neurology compared to non-neurology) | 0.013 | 0.272 | 0.097 | 0.763 | |
|  | Diabetes mellitus (type II) | Education subject | 0.031 |  |  |  | |
|  |  | Education subject (Chinese medicine compared to integrated medicine) | 0.018 | 0.530 | 0.314 | 0.897 | |
|  |  | Education subject (Conventional medicine compared to integrated medicine) | 0.031 | 0.520 | 0.287 | 0.943 | |
|  | Low plasma urate levels | Departments (Neurology compared to non-neurology) | 0.014 | 10.105 | 1.591 | 64.168 | |
|  | Physical inactivity | Age (years) | 0.000 | 1.278 | 1.113 | 1.467 | |
|  |  | Medical practice experience (years) | 0.015 | 0.867 | 0.773 | 0.972 | |
|  |  | Professional titles | 0.022 |  |  |  | |
|  |  | Professional titles (Chief physicians compared to resident physicians) | 0.016 | 0.322 | 0.128 | 0.811 | |
|  |  | Professional titles (Attending physicians compared to resident physicians) | 0.007 | 0.397 | 0.202 | 0.780 | |
| Antiparkinsonian medications | Levodopa | Professional titles | 0.016 |  |  |  | |
|  |  | Professional titles (Chief physicians compared to resident physicians) | 0.022 | 0.444 | 0.221 | 0.890 | |
|  |  | Professional titles (Attending physicians compared to resident physicians) | 0.008 | 0.408 | 0.210 | 0.795 | |
|  | Dopamine agonists | Professional titles | 0.031 |  |  |  | |
|  |  | Professional titles (Chief physicians compared to resident physicians) | 0.205 | 0.678 | 0.372 | 1.237 | |
|  |  | Professional titles (Attending physicians compared to resident physicians) | 0.009 | 0.453 | 0.251 | 0.817 | |
| Note: PD: Parkinson's disease; RBD: rapid eye movement sleep behaviour disorder. All surveyed items related to Parkinson's disease (PD) knowledge, including motor symptoms, non-motor symptoms, prodromal symptoms, risk and protective factors, and antiparkinsonian medications, were initially considered as dependent variables. However, only the regression analyses that were successfully constructed and met the statistical criteria for inclusion are presented in this table. Method = Forward Stepwise (Likelihood Ratio); Independent variables for the model: age (years), Medical practice experience (years), Categories of hospital, Departments (Neurology vs non-neurology), Education qualification, Education subject, Professional titles. | | | | | | |  |
